# Supplementary material for: Comparative Analyses of the Fecal Microbiome of Five Wild Black-Billed Capercaillie (Tetrao parvirostris) Flocks
Source: Animals (Basel). 2023 Mar 3;13(5):923. doi: 10.3390/ani13050923 (PMC10000248; doi:10.3390/ani13050923)
Supplement: Supplementary file 1 [file animals-13-00923-s001.zip › animals-2066884-supplementary.pdf]

Supplementary Material

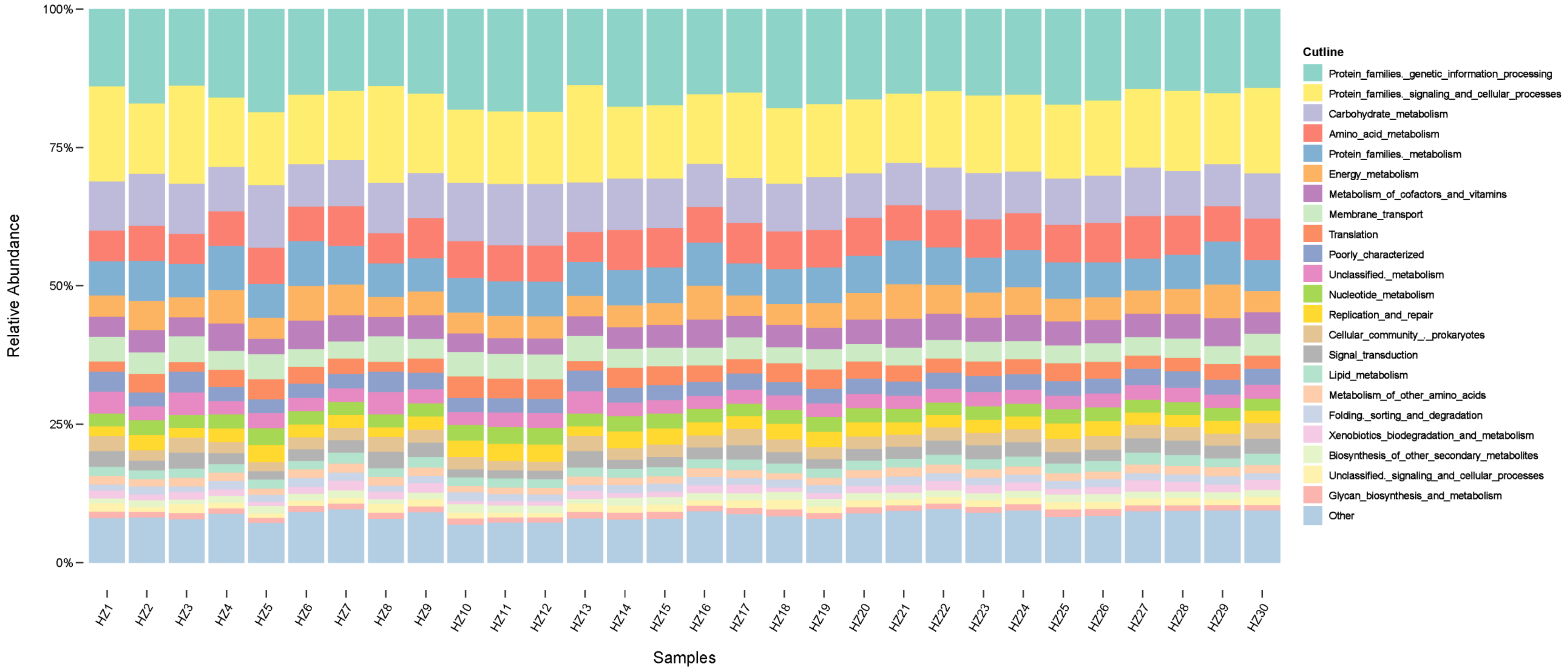

Figure S1 The relative abundance column cumulative plot of predicted KEGG function in each sample.
